# Genetic diversity and main functional composition of Lingzhi strains from main producing areas in China

**DOI:** 10.1186/s13568-021-01280-y

**Published:** 2021-08-21

**Authors:** Yuan-chao Liu, Xiao-cui Tang, Hui-ping Hu, Di-ling Chen, Yi-zhen Xie, Xiao-wei Liang, Xiang-min Li, Chun Xiao, Long-hua Huang, Qing-ping Wu

**Affiliations:** 1 School of Biology and Biological Engineering, South China University of Technology, Guangzhou, China; 2 Guangdong Provincial Key Laboratory of Microbial Safety and Health, State Key Laboratory of Applied Microbiology Southern China, Institute of Microbiology, Guangdong Academy of Sciences, Guangzhou, China; 3 Guangdong Yuewei Edible Fungi Technology Co., Ltd., Guangzhou, China

**Keywords:** Lingzhi, *Ganoderma lucidum*, Polysaccharides and triterpenoids, Monosaccharide, GBS technique, SNP, Phylogenetic tree

## Abstract

In this study, we used genotyping by sequencing (GBS) to examine the genetic diversity of 22 strains of Lingzhi and the quality differences in 15 fruit bodies of Lingzhi from different Chinese regions. The phylogenetic trees of 22 strains were constructed based on ITS (Internal transcribed spacer) and SNP (single nucleotide polymorphism). Moisture, ash, water-soluble extracts, alcohol-soluble extracts, polysaccharides, and triterpenoids from 15 fruit bodies of Lingzhi were detected and analyzed based on Chinese Pharmacopoeia and the US Pharmacopoeia references. Moreover, the monosaccharide composition of polysaccharides was studied using PMP-HPLC, and the effect of polysaccharides on the proliferation rate of splenocytes was investigated in vitro. The identification results of these strains by the phylogenetic trees which were constructed based on ITS sequences and SNPs showed that most of the strains applied in the main producing areas of Lingzhi in China were accurate except for a few inaccurate strains. The moisture, ash, water and alcohol soluble extractive, polysaccharide and triterpenoid content of all samples were meet the requirements of the Chinese Pharmacopoeia, while the polysaccharide and triterpenoid content of less than half of the samples meet the requirements of the U.S. Pharmacopoeia. The polysaccharide extracted from these samples have different effects on the proliferation rate of spleen cells. To sum up, this is the first study that reported on the differences in Lingzhi strains from the main producing areas in China. The quality of some fruit bodies did not meet the pharmacopeia requirements, and wrong strains were used in some production areas; thus, strains should be given special attention before legal processing.

**Supplementary Information:**

The online version contains supplementary material available at 10.1186/s13568-021-01280-y.

## Introduction

*Ganoderma lucidum* (also known as Lingzhi or *Ganoderma lingzhi* in China) is an edible and medicinal fungus that belongs to the genus *Ganoderma,* family Polyporaceae in Basidiomycete (Dai et al. 2013; Zhou et al. 2015). As a traditional Chinese herb, it has been applied for 2300 years to treat various human conditions. It can enrich the yin and nourish the kidney, relieve cough and asthma, prolong life, and support healthy energy (Paterson 2006; Li et al. 2012, 2016). The most abundant biologically active substances in Lingzhi are polysaccharides, nucleosides, triterpenoids, peptides, sterols, protein, and alkaloids (Sun et al. 2014). Polysaccharides and triterpenoids are considered to be the main medicinal components. Studies have shown that Lingzhi protects the liver; it has anti-tumor, anti-inflammatory, anti-virus, anti-oxidation, anti-aging, anti-radiation effect; it regulates the endocrine system, enhances immunity. Moreover, it can reduce blood glucose, reduce uric acid, blood lipids, and regulate gut microbiota (Tang et al. 2005b; Yan et al. 2010; Wasser 2014; Wang et al. 2015; Chang et al. 2017; Yan et al. 2017)

*Ganoderma spp* is globally distributed, being predominant in the tropical and subtropical regions, including China (Dai et al. 2013; Richter et al. 2015). Besides, Lingzhi has also been artificially cultivated in Zhejiang Longquan, Heilongjiang, Jilin, Hebei, Shandong, Anhui Huoshan, Jiangsu, Jiangxi, Hunan, Guizhou, Fujian, and Guangxi provinces (Zhao 1989; Zhang 2013), thus making China the global leading exporter of Lingzhi (Jin et al. 2016). According to the available statistics, the yield of Lingzhi and its spore powder was about 12 million tons in 2015, and the output value was 1.6 billion dollars, accounting for about 75% and 30% of global values. However, the quality of Lingzhi may vary based on differences in strain, production region, growing condition, cultivation techniques, and harvesting time (Lu et al. 2012; Chen et al. 2016; Zhao 2020). Thus, accurate identification and quality assessment of *G. lingzhi* are very important. Considering the uncertainty of the morphological characteristics of fruit body in different growth periods, molecular biological methods are commonly applied for quality assessment of *G. lingzhi* (Wang et al. 2011). According to related reports, ITS 2 (internal transcribed spacer 2) (Liao et al. 2015), ITS (Su et al. 2007), RAPD (random amplified polymorphic-DNA) (Wang et al. 2011), and SCAR sequence charactered amplified region) techniques (Xu et al. 2008) were used to identify *Ganoderma* (Lingzhi) fruit bodies of different origins. The results showed that these methods have certain effectiveness but could not distinguish between similar samples. Thus, a more accurate method should be developed.

Over the years, several high-throughput sequencing methods combined with bioinformatics analysis have been developed, which can free up tedious PCR workload and improve detection efficiency and accuracy. High-throughput sequencing techniques based on restriction enzyme digestion include GBS (Genotyping By Sequencing), RRLs (reduced-representation libraries), CRoPS (complexity reduction of polymorphic sequences), and RAD-Seq (restriction-site-associated DNA sequencing). GBS is a cost-effective approach widely applied in SNP (single nucleotide polymorphism) detection and genotyping research (Sonah et al. 2013). These techniques can be applied in molecular marker development and genotyping to model species with high-quality reference genomic sequences and the non-reference genomic species (Xiao et al. 2014).

In this study, we used the GBS technique to examine the genetic relationship of 22 strains of Lingzhi and ITS sequences. Moisture, ash, water-soluble extracts, alcohol-soluble extracts, polysaccharides, and triterpenoids of 15 fruit bodies of Lingzhi were detected and analyzed based on Chinese Pharmacopoeia and the US Pharmacopoeia references. Moreover, the monosaccharide composition of polysaccharides was studied using PMP-HPLC, and the effects of polysaccharides on the proliferation rate of splenocytes were investigated in vitro.

## Materials and methods

### DNA extraction of mycelium and ITS-PCR

The strains and the fruit bodies (part of) cultivated by the strain from the main producing areas of *G. lingzhi* in China were investigated. Fifteen fruit bodies and 22 strains of Lingzhi were collected (Additional file 1: Table S1). Strains were inoculated into the sterilized liquid fermentation medium (PD, autoclaved at 121 °C and 98 kPa for 20 min) in the shaker at 27 °C, 150 rpm for 9 days. Samples were then centrifuged at 8000 rpm to discard the medium, and mycelia were washed with pure water three times and prepared for use.

The TaKaRa MiniBEST Plant Genomic DNA Extraction Kit and PCR mix [Prime STAR Max Premix (2 ×) bought from Takara Biomedical Technology (Beijing) Co., Ltd.] were used for extracting total genomic DNA and PCR of 22 strains, respectively. The ITS primers and PCR protocol were applied as previously described (White et al. 1990). The primers synthesized and PCR products sequencing were obtained from Beijing Genomics Institute (BGI).

### Construction of phylogenetic tree based on ITS sequences

Parts of Sequences in this study used for phylogenetic analysis were downloaded from GenBank. Sequences were aligned using Clustal X (Thompson et al. 1997) and edited by Bioedit (Hall 1999). Phylogenetic analyses were performed with MEGA v7.0.26 (Kumar et al. 2016). A phylogenetic tree was constructed using the maximum likelihood method. Bootstrap values were calculated from 1000 replicates. Branches corresponding to partitions reproduced in less than 50% bootstrap replicates were collapsed. All positions containing gaps and missing data were used from the dataset.

### GBS sequencing and genetic structure analysis

Guangzhou Jidi’ao Biotechnology Co., Ltd preferred a GBS library construction and sequencing of 22 Lingzhi mycelia samples. The genome of each sample was digested with restriction endonuclease (GAATTC, CATG), and then T4 DNA ligase was used to ligate the adapter with a barcode. A small fragment library (250–550 bp) was constructed for double-ends sequencing by PE125. High-quality reads which were obtained by strict quality control, including removing the reads with N ratio of more than 10% and the low-quality reads, were mapped to the reference genomes with mem algorithm using BWA v0.7.12(Li and Durbin 2009); *Ganoderma lucidum* G.260125-1 was used as a reference genome (Chen et al. 2012), mapping parameter was -k 32–M. The results were marked using Picard v1.119 (Wysoker et al. 2014). Unified Genotyper module of the software GATK v3.4 (Mckenna et al. 2010) was used to handle the mapping file for Variant detection of multiple samples. The detected variants were filtered by Variant Filtration with filter parameters -Window4, -filter “QD < 4.0 || FS > 60.0 || MQ < 40.0”, - G_filter” GQ < 20”. ANNOVAR (Wang et al. 2010) was used to annotate the detected Variants. Plink (Purcell et al. 2007) and Frappe (Tang et al. 2005a) were used to infer the population structure within the different Lingzhi with different K values (from 2 to 10). Principal component analysis (PCA) was accomplished with R language (http://www.r-project.org/) based on the SNP between individuals. The phylogenetic tree was constructed by TreeBeST v1.9.2 (Vilella et al. 2009) with a neighbor-joining method based on the SNP. The samples were clustered to analyze the genetic relationship among the populations.

### Component detection and analysis

Moisture, ash, polysaccharides, and triterpenoids of samples from Lingzhi fruit bodies were detected using the latest version of Chinese Pharmacopoeia (Committee Chinese Pharmacopoeia 2015) and US (Convention the United States Pharmacopeial 2016); water and alcohol soluble extract was slightly modified based on the Chinese Pharmacopoeia. The colorimetric method by an ultraviolet spectrophotometry was used to determine the content of polysaccharides and total triterpenes at 490 nm and 546 nm, respectively. The phenol–Sulfuric Acid method was selected for the detection of polysaccharides, and Oleanolic acid was used as a reference substance for the detection of total triterpenes. Data analysis and graphs constructed were performed using Excel (Microsoft 365).

### Effects of polysaccharides on proliferation ability of splenocytes in vitro

Balb/c male nude mice, 8 weeks old, weighing 20–25 g, were obtained from Guangdong Medical Laboratory Animal Center, Guangzhou, China. All the animals were housed in the SPF Animal Laboratory of Guangdong Institute of Microbiology, kept an environment with a temperature of 25 ± 1 °C, relative humidity of 55 ± 1%, and a light/dark cycle of 12/12 h. All experimental protocols were approved by the Animal Ethics Committee of Guangdong Institute of Microbiology, and all experimental procedures were conformed by the National Institutes of Health Guide for the Care and Use of Laboratory Animals. Spleens were removed from mice and immersed in PBS buffer, cut into pieces with scissors, gently ground with a syringe piston. The cells suspension was obtained using a 200-mesh cell filter. The cells filtrate was then centrifuged for 5 min at RCF 250×*g*, after which the supernatant was discarded. The rest was mixed with 1 ml ACK lysate (room temperature) to resuspended cells at room temperature for 5 min. Cells were then mixed with 7 ml precooling PBS, centrifuged for 5 min at 250×*g*, and washed twice with cooling PBS.

The effects of polysaccharides on the proliferation ability of splenocytes in vitro were examined using a previously described approach (Li et al. 2019) with a slight modification. Spleen cells (6 × 10^6^ cells/mL, 100 µL) were seeded onto 96-well tissue culture plates. After reaching an 80% confluence, cells were incubated in polysaccharide (10 µg) dissolved in 100 µl DMEM (10% FBS, 100 U/ml penicillin/streptomycin) and at 37 °C and 5% CO_2_ for 72 h. MTS method was used to measure the OD value at 490 nm by a microplate reader (MUITISKAN GO, Thermo Scientific). All data obtained were analyzed by Kruskal–Wallis test performed with R software, version 3.5.3 (R Foundation, Vienna, Austria) (Kaur et al. 2020), p-values of less than 0.05 were considered to indicate statistical significance.

## Results

### Sequencing data and phylogenetic tree

The ITS-rDNA sequences in this study have been uploaded to NCBI, and the GenBank Accession Numbers were MN911326–MN911347. Other sequences, including the outgroup *Amauroderma rude,* were download from NCBI. Twenty-two strains were sampled for GBS. A total of 3.649 Tb of clean data and 3.567 Tb of HQ clean data after filtration were generated. The effective tags coverage in the genome was 0.19% (Additional file 1: Tables S2–S4). The raw data of the GBS sequence has been uploaded to NCBI, and the SRA Accession Number is PRJNA600664.

**Fig. 1:**
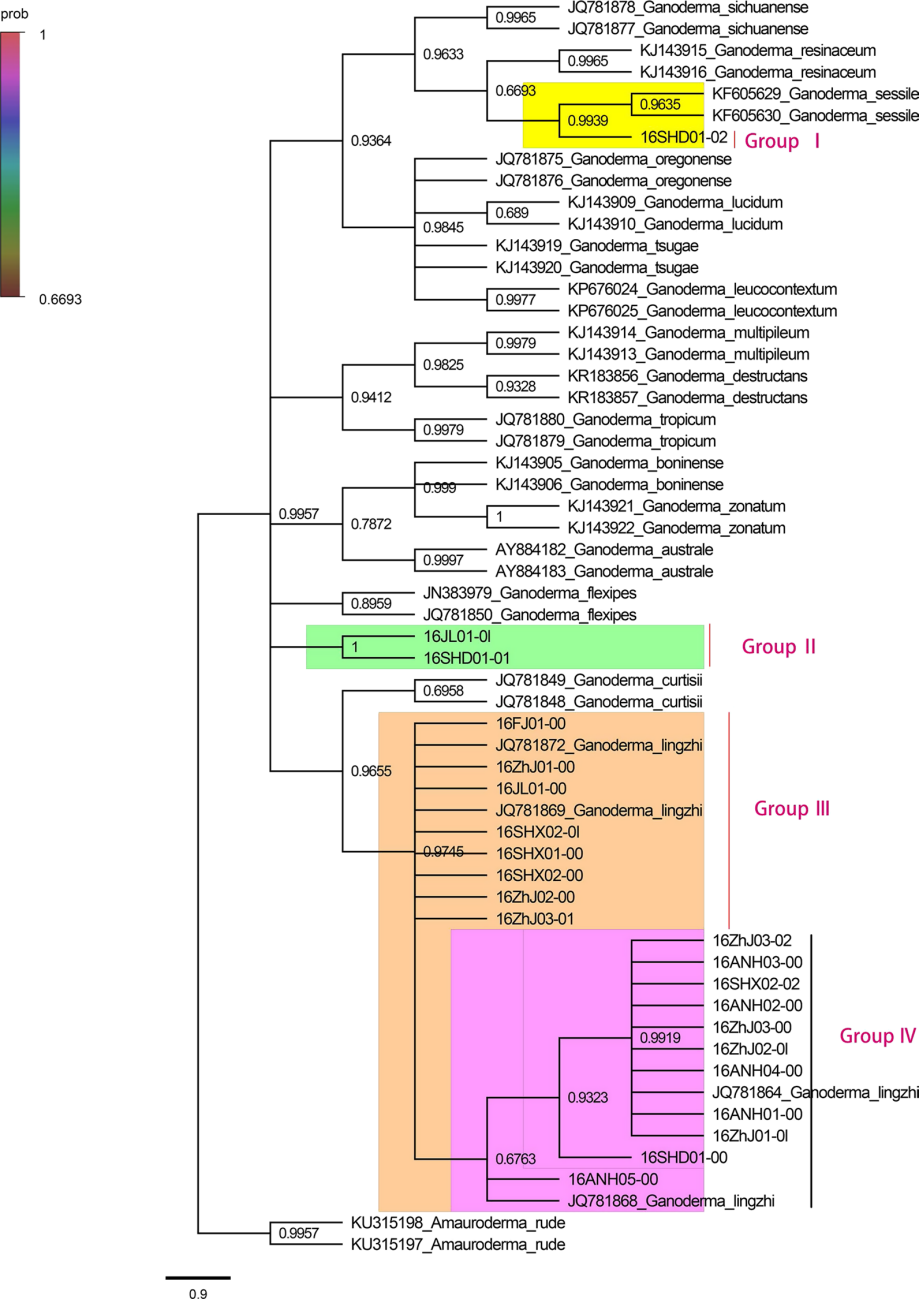
Phylogenetic tree of 22 lingzhi strains based on ITS sequences

The phylogenetic tree constructed based on ITS-rDNA sequences revealed that 22 Lingzhi strains could be divided into four categories. Among these strains, 16SHD01-02 (Group I) from Liaocheng city, Shandong Province, were clustered in the same category with *Ganoderma sessile* (and identified as *Ganoderma sessile)*, which is consistent with the reference genome comparison (Additional file 1: Table S5). 16JL01-0l from Jiaohe city, Jilin Province, and 16SHD01-01 from Liaocheng city, Shandong Province, were individually clustered in one small category (Group II). The remaining 19 strains were clustered together with *Ganoderma lingzhi*, and were identified as *Ganoderma lingzhi*. Among these strains, 16ZHJ03-02, 16SHX02-02, 16ANH02-00, 16ZHJ01-01, 16ZHJ03-00, and 16ANH01-00 were closely related and clustered in one branch (Group IV); all showed high spore powder production ability. 16SHX02-00, 16ZHJ03-01, 16ZHJ01-00, 16ZHJ02-00, 16FJ01-00, and 16SHX01-00 were closely related and clustered in another branch (Group III) (Fig. 1).

**Fig. 2:**
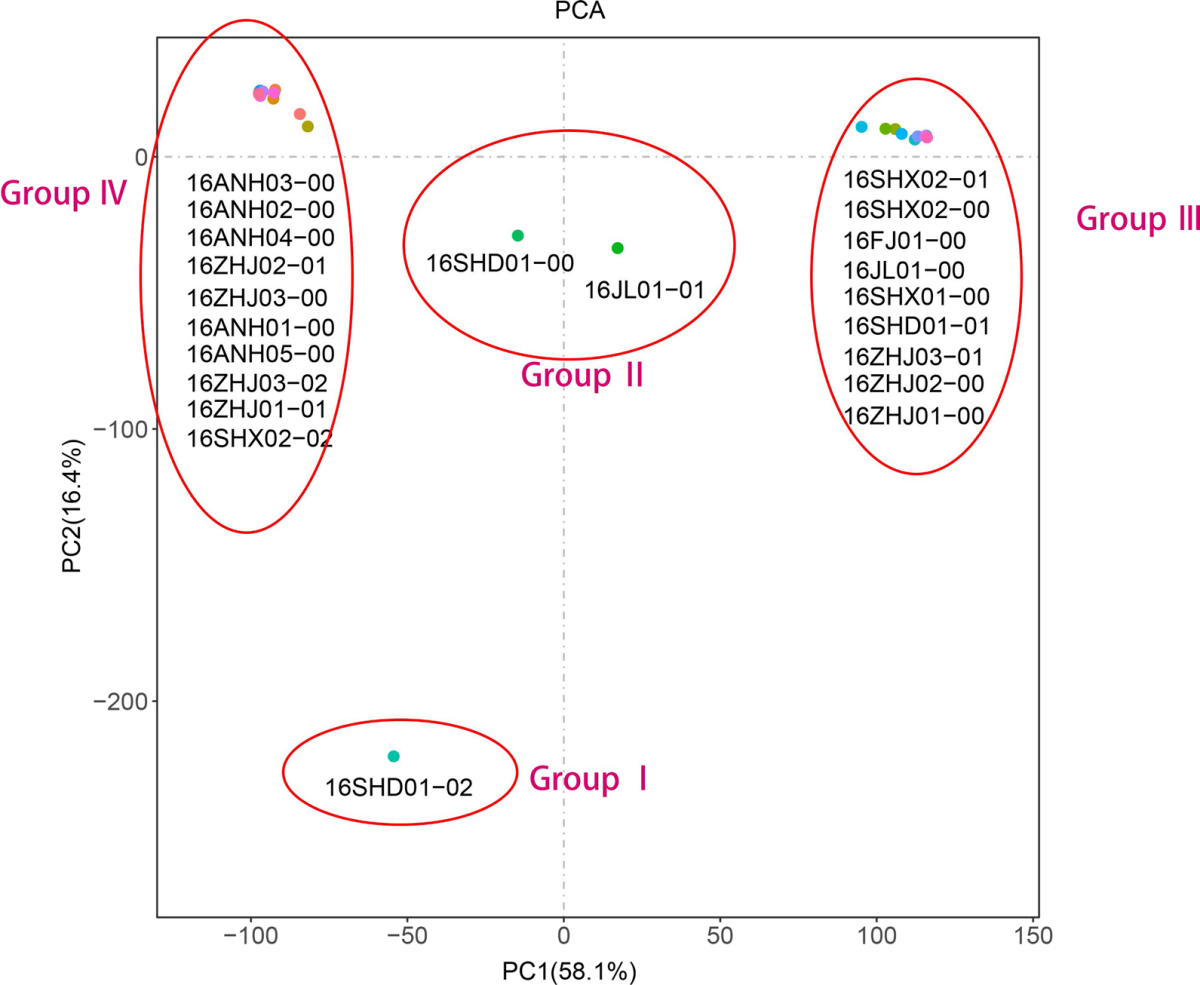
Principal component analysis of 22 lingzhi strains based on SNP

The principal component analysis (PCA) demonstrated that the first component could differentiate the Group I from Group II, Group III and Group IV, the Group II, Group III, and Group IV was in the second component, but they could be separated significantly. The first and second components explained 58.1% and 16.4% of the SNP variances, respectively (Fig. 2). Altogether, these results indicated that Group II, Group III, and Group IV were similar but were significantly different from Group I; this result was consistent with the phylogenetic tree based on ITS sequences, revealing that 16SHD01-02 was not *G. lingzhi*.

**Fig. 3:**
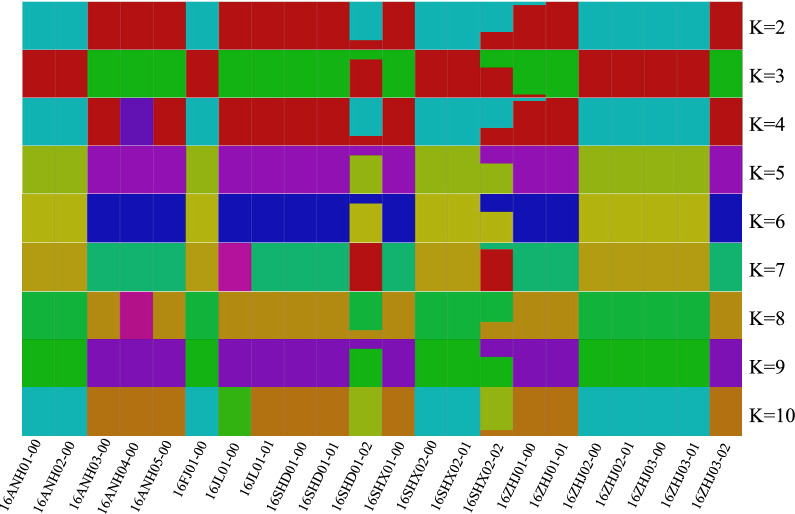
Population structure analysis of 22 lingzhi strains based on SNP

**Fig. 4:**
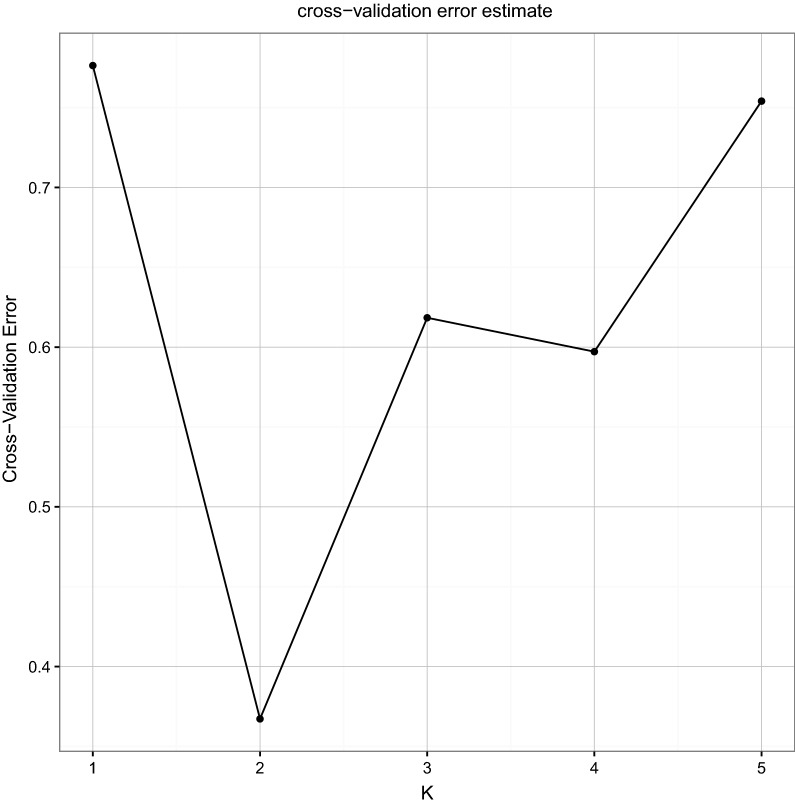
Analysis of the cross validation error corresponding to different K values

Next, the population structure among the samples with different values for K (from 2 to 10) was analyzed (Fig. 3). The K value was determined based on the cross-validation error rate, and the K value with the lowest cross-validation error rate was the optimal K value (Fig. 4). When K was 2, the cross-validation error rate was the lowest, which indicated that the best classification strategy for the population was divided into two subgroups.

**Fig. 5:**
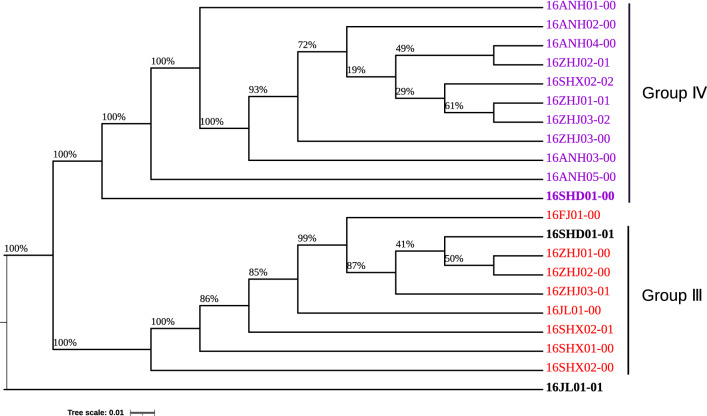
Phylogenetic tree of 22 lingzhi strains based on SNP

As 16SHD01-02 was not considered Lingzhi, the strain was removed from the group in the following phylogenetic tree analysis. Based on the effective SNPs (Additional file 1: Table S6 and Additional file 2: Table S7), the Neighbor-Joining (NJ) phylogenetic trees of the Lingzhi strains across China were constructed. All Lingzhi strains formed three different clades (Fig. 5; the two main branches strains almost contained all Lingzhi strains, 16ANH01-00, 16ANH02-00, 16ANH03-00, 16ANH04-00, 16ANH05-00, 16SHX02-02, 16ZHJ01-01, 16ZHJ03-00, 16ZHJ02-01, 16SHD01-00. 16ZHJ03-02 formed one of the main branches, which was similar to the group IV, while 16FJ-01-00, 16JL01-00, 16SHD01-01, 16SHX01-00, 16SHX02-00, 16SHX02-01, 16ZHJ01-00, 16ZHJ03-01and 16ZHJ02-00 formed another main branch, which was similar to the group III. However, 16JL-01-01 formed a single branch alone.

### The result of the component determination

#### Moisture and ash content

According to the 2015 edition of the Chinese Pharmacopoeia and the US, moisture content of *G. lucidum* should not exceed 17%. In this study, the moisture contents of fruit bodies from 15 samples of Lingzhi were low. The lowest moisture content was observed in 16SHX02-00 (1.47%), and the highest content in 16ZHJ03-00 (7.28%).

In addition, according to the Chinese Pharmacopoeia, the ash content of *G. lucidum* should not be more than 3.2%, while according to the US Pharmacopoeia, ash content should go above 4%. In this study, the ash contents of fruit bodies from 15 samples of Lingzhi did not exceed the specified limit (Table 1).

**Table 1:** The content of moisture and total ash

Sample	Moisture/%	Total ash/%
16ANH01-00	4.83	1.927
16ANH02-00	3.28	1.928
16FJ01-00	2.32	1.928
16SHD01-00	3.02	1.928
16SHD01-01	6.86	1.925
16SHD01-02	4.03	1.927
16SHX01-00	1.93	1.929
16SHX02-00	1.47	1.929
16SHX02-02	3.93	1.927
16ZHJ01-00	2.52	1.928
16ZHJ01-01	1.97	1.928
16ZHJ02-00	7.12	1.925
16ZHJ03-00	7.28	1.925
16ZHJ03-01	2.61	1.928
16ZHJ03-02	5.88	1.926

#### Water and alcohol soluble extractive

The result of water and alcohol extractives from fruit bodies are shown in Table 2. According to the 2015 edition of the Chinese and the US Pharmacopoeia, the water-soluble extractive from fruit bodies of *G. lucidum* should not be less than 3%. Our results showed that the water-soluble extractive of fruit bodies from 15 samples of Lingzhi exceeded the specified limit; the lowest water-soluble extractive was observed in 16ZHJ03-02 (6.5%), and the highest was in 16SHD01-00 (16.43%).

**Table 2:** The results of water soluble and alcohol soluble extract content

Sample	Water-soluble extract content (%)	Alcohol-soluble extract content (%)
16ANH01-00	8.05	4.2
16ANH02-00	7.93	3.41
16FJ01-00	8.32	2.45
16SHD01-00	16.43	6.39
16SHD01-01	8.56	3.4
16SHD01-02	10.62	3.46
16SHX01-00	9.47	3.64
16SHX02-00	9.31	3.19
16SHX02-02	13.33	3.93
16ZHJ01-00	7.38	2.74
16ZHJ01-01	7.82	3.26
16ZHJ02-00	9.03	2.61
16ZHJ03-00	9.27	2.88
16ZHJ03-01	8.62	2.69
16ZHJ03-02	6.5	2.57

The Chinese Pharmacopoeia did not stipulate the limit of alcohol-soluble extractive of *G. lucidum*, while US Pharmacopoeia indicated that the alcohol-soluble extractive of *G. lucidum* should not be less than 2%. The results showed that the alcohol-soluble extractive of fruit bodies from15 samples all met the specified limit; the highest alcohol-soluble extractive was 6.39% in 16SHD01-00, and the lowest was 2.45% in 16FJ01-00.

#### Polysaccharides content

According to the Chinese Pharmacopoeia, the polysaccharides content of *G. lucidum* should not be < 0.9%. Table 3 shows that the polysaccharides contents of fruit bodies from 15 samples of Lingzhi determined by the colorimetric method greatly varied from 1.04 to 3.19%; the highest polysaccharides content was seen in 16SHX02-02 (3.19%) followed by 16SHX01-00 (2.23%), 16ZHJ01-01 (1.91%), 16ZHJ03-00 (1.89%), and 16ZHJ02-00 (1.75%), respectively. The polysaccharides contents determined by HPLC method varied significantly from 0.72 to 2.51%; the lowest polysaccharides content was seen in 16SHX02-00 (0.72%), and the highest was 2.51% in 16SHX02-02, followed by 16ZHJ03-00, 16ZHJ01-01, 16SHX01-00, and 16ZHJ02-00 (1.95%, 1.87%, 1.65%, and 1.53%, respectively).

**Table 3:** The results of polysaccharides content by colorimetric, HPLC and PMP-HPLC method

Sample	Polysaccharides content (%)		
	Colorimetric method	HPLC method	PMP-HPLC method
16ANH01-00	1.72	1.26	0.83
16ANH02-00	1.55	1.43	0.47
16FJ01-00	1.07	0.83	0.51
16SHD01-00	1.45	1.16	0.95
16SHD01-01	1.07	0.91	0.46
16SHD01-02	1.18	0.98	0.33
16SHX01-00	2.23	1.65	0.81
16SHX02-00	1.04	0.72	0.57
16SHX02-02	3.19	2.51	0.9
16ZHJ01-00	1.14	0.82	0.51
16ZHJ01-01	1.91	1.87	0.5
16ZHJ02-00	1.75	1.53	0.55
16ZHJ03-00	1.89	1.95	0.56
16ZHJ03-01	1.41	0.99	0.66
16ZHJ03-02	1.41	1.35	0.39

According to the US Pharmacopoeia, polysaccharides content should not be less than 0.7% by PMP-HPLC method. The monosaccharide composition of these polysaccharides of fruit bodies from 15 samples of Lingzhi was mainly glucose after comparing and analyzing by PMP-HPLC method. The monosaccharide content of polysaccharides extracted from 16FJ01-00, 16SHD01-01, 16SHD01-02, 16SHX01-00, 16SHX02-00, 16ZHJ01-00, 16ZHJ01-01, 16ZHJ02-00, 16ZHJ03-01, and 16ZHJ03-02 from high to low were glucose, galactose, mannose, glucuronic acid, and fucose. The monosaccharide content of polysaccharides extracted from 16ANH01-00 and 16ANH02-00 from high to low were glucose, mannose, galactose, glucuronic acid, and fucose. The monosaccharide content of polysaccharides extracted from 16SHD01-00, 16SHX02-02, and 16ZHJ03-00 from high to low were glucose, mannose, galactose, fucose, and glucuronic acid from high to low. As shown in Table 3, the polysaccharides contents of 15 samples of Lingzhi were different in the range of 0.33–0.95%, the lowest polysaccharides content was 0.33% in 16SHD01-02, and the highest polysaccharides content was 0.95% in 16SHD01-00.

**Fig. 6:**
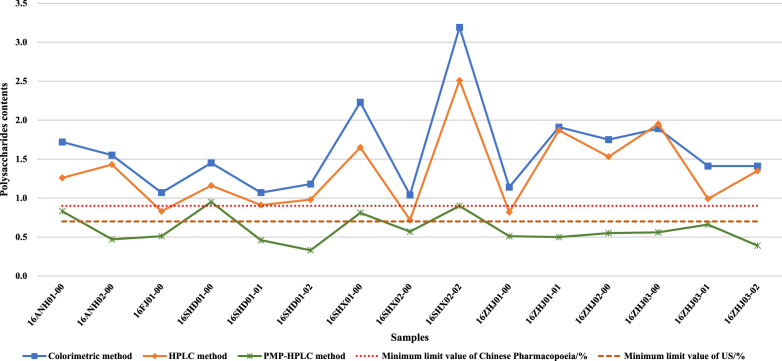
Polysaccharides contents extract from fruit body of 15 lingzhi

As shown in Fig. 6, the polysaccharides content extracted from these samples was quite different, which might be related to regions and growing climatic conditions. The detection method can affect the value, but the trend of the polysaccharide content of each sample was almost the same.

#### Triterpenoids content

According to the Chinese Pharmacopoeia, the triterpenoids content of fruit bodies from *G. lucidum* should not be less than 0.5%. Table 4 shows that the triterpenoids content of fruit bodies from 15 samples of Lingzhi determined by the colorimetric method was different in the range of 0.70–1.74%. The lowest triterpenoids content was seen in 16FJ01-00 (0.70%), and the highest in 16SHD01-00 (1.74%), followed by 16ANH01-00, 16ANH02-00, 16SHX02-02 and 16SHD01-01 (1.24%, 1.10%, 0.96% and 0.94%, respectively).

**Table 4:** The results of triterpenoids content by colorimetric and HPLC method

Sample	Triterpenoids content (%)	
	Colorimetric method	HPLC method
16ANH01-00	1.24	0.33
16ANH02-00	1.1	0.44
16FJ01-00	0.7	0.17
16SHD01-00	1.74	0.05
16SHD01-01	0.94	0.63
16SHD01-02	0.94	0.79
16SHX01-00	0.83	0.3
16SHX02-00	0.78	0.17
16SHX02-02	0.96	0.43
16ZHJ01-00	0.73	0.22
16ZHJ01-01	0.9	0.27
16ZHJ02-00	0.71	0.26
16ZHJ03-00	0.86	0.29
16ZHJ03-01	0.73	0.25
16ZHJ03-02	0.84	0.12

According to the US Pharmacopoeia, the triterpenoids content should not be less than 0.3%. Triterpenoids content determined by the HPLC method was different in the range of 0.05–0.79%; the lowest content was detected in 16SHD01-00 (0.05%) and the highest in 16SHD01-02 (0.79%). Based on the US Pharmacopoeia reference, only 16SHD01-01, 16SHD01-02, 16SHX01-00, 16SHX02-02, 16ANH01-00, and 16ANH02-00 met the requirement.

**Fig. 7:**
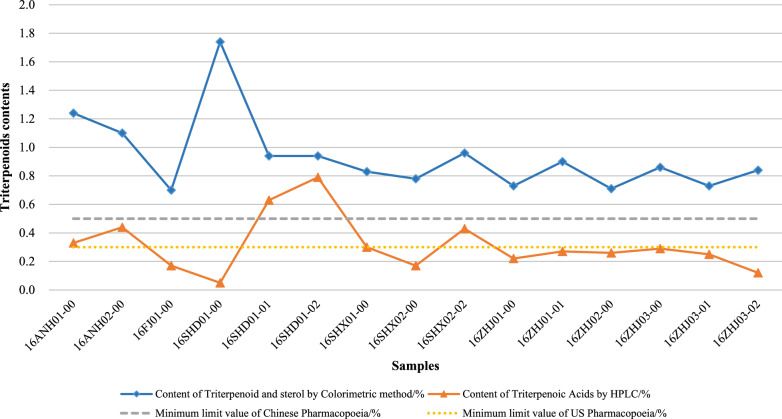
Triterpenoids contents extract from fruit body of 15 lingzhi

As seen in Fig. 7, triterpenoids content extracted from these samples was different. Also, the results of some samples by different detection methods varied greatly. The results obtained by the method of the US Pharmacopoeia were generally low, and most of them did not meet the quality requirements.

### Effect of splenic lymphocyte proliferation

**Fig. 8:**
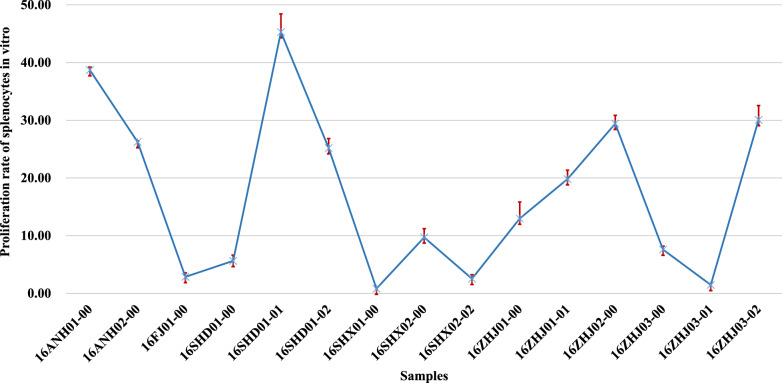
Proliferation rate of splenocytes in vitro treated with polysaccharides extracted from fruit bodies of 15 lingzhi

The proliferation rate of splenocytes treated with polysaccharides extracted from 15 samples of Lingzhi varied from 0.82 to 45.29% (Fig. 8); the highest proliferation rate was 45.29% from 16SHD01-01, followed by 16ANH01-00, 16ZHJ03-02, 16ZHJ02-00, 16ANH02-00, 16SHD01-02 (38.69%, 30.06%, 29.41%, 26.23%, 25.17%, respectively). 16SHX01-00 had the lowest proliferation rate on splenocytes (0.82%) (Table 5), the variance of the result was not homogeneous (P = 0.0124 < 0.1), then Kruskal–Wallis test was conducted, which reveal that most of the samples have significant differences **(**Additional file 3: Table S8).

**Table 5:** Effects of 15 kinds of Lingzhi polysaccharides on the proliferation of splenocytes in vitro

Sample	Proliferation rate/%					Amount of polysaccharide/ug
	Value1	Value2	Value3	Mean value	SD	
16ANH01-00	38.94	39.18	37.96	38.69	0.53	10
16ANH02-00	25.99	26.48	26.23	26.23	0.2	10
16FJ01-00	2.78	2.04	3.75	2.86	0.7	10
16SHD01-00	4.49	6.93	5.46	5.63	1	10
16SHD01-01	47.00	47.98	40.89	45.29	3.14	10
16SHD01-02	27.45	23.54	24.52	25.17	1.66	10
16SHX01-00	1.07	0.82	0.58	0.82	0.2	10
16SHX02-00	8.64	11.82	8.64	9.7	1.5	10
16SHX02-02	2.04	2.04	3.51	2.53	0.69	10
16ZHJ01-00	16.95	10.35	11.57	12.96	2.87	10
16ZHJ01-01	17.68	20.37	21.35	19.8	1.55	10
16ZHJ02-00	30.87	27.45	29.90	29.41	1.44	10
16ZHJ03-00	6.93	7.42	8.40	7.58	0.61	10
16ZHJ03-01	1.31	1.80	1.31	1.47	0.23	10
16ZHJ03-02	33.56	28.43	28.19	30.06	2.48	10

## Discussion

*G. lingzhi* is a large, dark mushroom that promotes health and longevity. Its quality can be affected by many factors such as type of strains, cultivation medium, environment, and artificial management. In this study, we investigated the strains and fruit bodies from the main production areas of *G. lingzhi* in China using genotyping by sequencing (GBS). The quality of *G. lingzhi* from each producing area was examined by analyzing genetic diversity, the content of polysaccharides and triterpenoids, and the proliferation rate of splenic lymphocytes treated with polysaccharide extracts from fruit bodies. We found differences in appearance (pileus) of *G. lingzhi* obtained from different areas. The phylogenetic tree based on ITS sequences revealed the genetic relationship of these Lingzhi strains. 16SHD01-02 was different from other strains, as its sequences match rate was only 16.82% with the reference genome (Chinese Pharmacopoeia and US Pharmacopoeia). The principal component analysis (PCA) and the phylogenetic tree based on SNP obtained similar results. A cluster of 16SHD01-01 and 16JL01-01 in the phylogenetic tree showed a difference, which may be related to different approaches used to construct the phylogenetic trees. In some studies (Escudero et al. 2014, Wong et al. 2015), the maximum likelihood (ML) method was used to construct phylogenetic trees based on SNPs. However, when we tried this method, the software presented an error prompt and could not complete the phylogenetic tree construction. After testing, it was found that problem was 16SHD01-02, as its genetic relationship was too far from other strains, which was consistent with PCA analysis. Finally, it was identified as *G sessile* by phylogenetic tree based on ITS. This made it clear that there was the wrong strain in the cultivation of Lingzhi.

The components extracted from fruit bodies may be greatly affected by the extraction method. Several polysaccharide extraction methods from *G. lingzhi*, such as hot water extraction, organic solvent extraction, enzymatic extraction, ultrasonic extraction, and microwave extraction, have been reported (Zhu et al. 2017; Shao et al. 2020). Methods for extracting triterpenoids mainly rely on spectrophotometry and high-performance liquid chromatography (Xu 2014). Some studies suggested that different extraction and detection methods may lead to different results (Yang et al. 2019). Therefore, scientific and accurate methods are necessary to assess the quality of Lingzhi.

In this study, the results of the determination of extract components from the fruit body of Lingzhi revealed that the content of moisture, ash, water-soluble extractive met the specification of Chinese Pharmacopoeia and US. Moreover, the monosaccharide composition of polysaccharides was studied using PMP-HPLC, and the effects of polysaccharides on the proliferation rate of splenocytes were investigated in vitro. The polysaccharides extracted from the fruit body by colorimetric method met the specification of the Chinese Pharmacopoeia. However, only 16SHX02-02, 16SHX01-00, 16ANH01-00, 16SHD01-00 met the polysaccharides content specification of the US Pharmacopoeia as differences in measurement methods. The same phenomenon was observed in the results of triterpenoids content; only 16SHD01-01, 16SHD01-02, 16SHX01-00, 16SHX02-02, 16ANH01-00, and 16ANH02-00 no less than the specification of the US Pharmacopoeia in triterpenoids content, which revealed that the measurement method had a huge impact on the results. Among the samples, polysaccharides extract from 16SHD01-01, 16SHD01-02, 16ANH01-00, and 16ANH02-00, which were at the same concentration, showed significant proliferation of splenocytes in vitro.

The content of polysaccharides is usually used as an important indicator of the quality of *G. lingzhi*. However, the results revealed no correlation between polysaccharide content and its activity; thus, it may be inadequate to focus only on the polysaccharide content and ignore its activity. Therefore, we think that the evaluation of the quality of *G. lingzhi* requires a comprehensive consideration, as the activity of the active ingredient and its content may be equally important for processors and consumers.

## Supplementary Information

**Additional file 1: Table S1.** Collecting information of Lingzhi sample(strains and fruit body). **Table S2.** Table of Genomic electronic digestion assessment statistics. **Table S3. **Table of base information before and after filter. **Table S4.** Statistics table of reads filter information. **Table S5.** HQ clean Reads vs. Reference Genomes. **Table S6.** Statistical of SNP in samples’ chromosome.

**Additional file 2.** Statistics of annotation results for each sample.

**Additional file 3.** The homogeneity of variance test and Kruskal-Wallis test of the spleen cell proliferation rate of each sample.

## Data Availability

The data involved in this article can be found in the main manuscript and supplementary data, and the sequencing data have been uploaded to NCBI.

## Abbreviations

GBS: Genotyping by sequencing
RRL: Reduced-representation libraries
CRoPS: Complexity reduction of polymorphic sequences
RAD-Seq: Restriction-site-associated DNA sequencing
SNP: Single nucleotide polymorphism
GLIS: The active polysaccharides extracted from Lingzhi
RAPD: Random Amplified Polymorphic DNA
ITS: Internal transcribed spacer
ISSR: Inter-simple sequence repeat
SCAR: Sequence characterized amplified regions
SRAP: Sequence-related amplified polymorphism
RFLP: Restriction fragment length polymorphism
AFLP: Amplified fragment length polymorphism
HPLC: High performance liquid chromatography
PMP: 1-Pheny-3-methyl-5-pyrazolone
RPM: Revolution(s) per minute
PCA: Principal component analysis
PBS: Phosphate buffered saline
