# Supplementary material for: Genetic diversity and main functional composition of Lingzhi strains from main producing areas in China
Source: AMB Express. 2021 Aug 21;11:119. doi: 10.1186/s13568-021-01280-y (PMC8379333; doi:10.1186/s13568-021-01280-y)
Supplement: Supplementary file 1 — Additional file 1: Table S1. Collecting information of Lingzhi sample(strains and fruit body). Table S2. Table of Genomic electronic digestion assessment statistics. Table S3. Table of base information before and after filter. Table S4. Statistics table of reads filter information. Table S5. HQ clean Reads vs. Reference Genomes. Table S6. Statistical of SNP in samples’ chromosome. [file 13568_2021_1280_MOESM1_ESM.docx]

Additional file 1: Table S1. Collecting information of Lingzhi sample(strains and fruit body)

| Sample name | Sample number | Producing area | strains | Fruit body | Collecting date |
| --- | --- | --- | --- | --- | --- |
| Dapianlingzhi | 16SHD01-00 | Jingdangpu village, Guan county, Liao city, Shandong province | * | * | 2016.9.01 |
| Hanzhi | 16SHD01-01 | Jingdangpu village, Guan county, Liao city, Shandong province | * | * | 2016.9.01 |
| Meiguodapian | 16SHD01-02 | Jingdangpu village, Guan county, Liao city, Shandong province | * | * | 2016.9.01 |
| Hanzhi No.2 206 | 16ZHJ01-00 | Shuangxi village, Chengbei county, Longquan city, Zhejiang province | * | * | 2016.7.25 |
| Hanzhi No.1 | 16ZHJ01-01 | Shuangxi village, Chengbei county, Longquan city, Zhejiang province | * | * | 2016.7.25 |
| Hanzhi No.2 203 | 16ZHJ02-00 | Xiaojiao village, Longquan city, Zhejiang province | * | * | 2016.7.26 |
| Hanzhi No.1 | 16ZHJ02-01 | Xiaojiao village, Longquan city, Zhejiang province | * |  | 2016.7.26 |
| Hanzhi No.1 119 | 16ZHJ03-00 | Shilong village, Longquan city, Zhejiang province | * | * | 2016.7.26 |
| Longzhi No.2 | 16ZHJ03-01 | Shilong village, Longquan city, Zhejiang province | * | * | 2016.7.26 |
| Hunong No.1 119 | 16ZHJ03-02 | Shilong village, Longquan city, Zhejiang province | * | * | 2016.7.26 |
| Hanzhi No.2 206 | 16FJ01-00 | Jixi village, Shuibeijie town, Pucheng county, Fujian province | * | * | 2016.7.26 |
| Hanzhi No.8 | 16SHX01-00 | Wudu town, Chenggu county, Hanzhong city, Shanxi province | * | * | 2016.7.31 |
| Longzhi No.1 | 16SHX02-00 | Hanzhong city, Shanxi province | * | * | 2016.8.01 |
| Hanzhi No.3 | 16SHX02-01 | Huangan town, Yang county, Hanzhong city, Shanxi province | * |  | 2016.8.01 |
| Hunong No.1 | 16SHX02-02 | Huangan town, Yang county, Hanzhong city, Shanxi province | * | * | 2016.8.01 |
| Chizhi No.9 | 16ANH01-00 | Meishan, Shahe, Jinzhai, Anhui province | * | * | 2016.8.02 |
| Hunong No.1 s | 16ANH02-00 | Suban, Jinzhai, Anhui province | * | * | 2016.8.03 |
| Regrowth of Hunong No.1 | 16ANH03-00 | Lanshui, Jinzhai, Anhui province | * |  | 2016.8.02 |
| Lingzhi | 16ANH04-00 | Shahe, Jinzhai, Liu'an, Anhui province | * |  | 2016.8.03 |
| Lingzhi | 16ANH05-00 | Shahe, Jinzhai, Liu'an, Anhui province | * |  | 2016.8.03 |
| Hanzhi | 16JL01-00 | Huangsongdian, Jiaohe, Jilin province | * |  | 2016.8.30 |
| Ribenlingzhi | 16JL01-01 | Huangsongdian, Jiaohe, Jilin province | * |  | 2016.8.30 |

Additional file 1: Table S2. Table of Genomic electronic digestion assessment statistics

| **Genome Size** | **All Tags number** | **All Tags length** | **All Tags coverage** | **All RF ave-length** | **Effective Tags number** | **Effective Tags length** | **Effective Tags coverage** | **Effective RF ave-length** |
| --- | --- | --- | --- | --- | --- | --- | --- | --- |
| 43292593 | 15226 | 2162092 | 0.0499 | 5559 | 590 | 83780 | 0.0019 | 393 |

Additional file 1: Table S3. Table of base information before and after filter

| **sample** | **Before Filter** | | | | | **After Filter** | | | | |
| --- | --- | --- | --- | --- | --- | --- | --- | --- | --- | --- |
|  | **Clean Data(bp)** | **Q20(%)** | **Q30(%)** | **N(%)** | **GC(%)** | **HQ Clean Data(bp)** | **Q20(%)** | **Q30(%)** | **N(%)** | **GC(%)** |
| 16ANH01-00 | 46335452 (100.00%) | 43809430 (94.55%) | 40462989 (87.33%) | 143 (0.00%) | 25282359 (54.56%) | 45191880 (97.53%) | 42918717 (94.97%) | 39727273 (87.91%) | 111 (0.00%) | 24676055 (54.60%) |
| 16ANH02-00 | 163273304 (100.00%) | 154629203 (94.71%) | 143189404 (87.70%) | 969 (0.00%) | 88597119 (54.26%) | 158680707 (97.19%) | 151081754 (95.21%) | 140228790 (88.37%) | 348 (0.00%) | 86211022 (54.33%) |
| 16ANH03-00 | 76636264 (100.00%) | 72445120 (94.53%) | 66891339 (87.28%) | 181 (0.00%) | 41644300 (54.34%) | 74941520 (97.79%) | 71160350 (94.95%) | 65851371 (87.87%) | 103 (0.00%) | 40742654 (54.37%) |
| 16ANH04-00 | 220906844 (100.00%) | 208614228 (94.44%) | 192346871 (87.07%) | 980 (0.00%) | 120068956 (54.35%) | 216360382 (97.94%) | 205243050 (94.86%) | 189668770 (87.66%) | 389 (0.00%) | 117633243 (54.37%) |
| 16ANH05-00 | 171457332 (100.00%) | 162487491 (94.77%) | 150374859 (87.70%) | 599 (0.00%) | 93159587 (54.33%) | 167821166 (97.88%) | 159648840 (95.13%) | 148025116 (88.20%) | 414 (0.00%) | 91232342 (54.36%) |
| 16FJ01-00 | 140084136 (100.00%) | 132359403 (94.49%) | 122148517 (87.20%) | 775 (0.00%) | 76201118 (54.40%) | 136952661 (97.76%) | 129961975 (94.90%) | 120195844 (87.76%) | 332 (0.00%) | 74535964 (54.42%) |
| 16JL01-00 | 180141200 (100.00%) | 170660458 (94.74%) | 157883960 (87.64%) | 946 (0.00%) | 98254437 (54.54%) | 176288065 (97.86%) | 167658176 (95.10%) | 155403380 (88.15%) | 404 (0.00%) | 96210428 (54.58%) |
| 16JL01-01 | 284291668 (100.00%) | 268111246 (94.31%) | 246951052 (86.87%) | 1391 (0.00%) | 153707639 (54.07%) | 278274977 (97.88%) | 263668134 (94.75%) | 243436555 (87.48%) | 646 (0.00%) | 150506826 (54.09%) |
| 16SHD01-00 | 65388728 (100.00%) | 61890583 (94.65%) | 57286601 (87.61%) | 381 (0.00%) | 35129553 (53.72%) | 63328614 (96.85%) | 60240656 (95.12%) | 55893464 (88.26%) | 154 (0.00%) | 34051394 (53.77%) |
| 16SHD01-01 | 231706228 (100.00%) | 218508372 (94.30%) | 201224534 (86.84%) | 1031 (0.00%) | 125646124 (54.23%) | 226953512 (97.95%) | 215016946 (94.74%) | 198471406 (87.45%) | 475 (0.00%) | 123109198 (54.24%) |
| 16SHD01-02 | 191151880 (100.00%) | 179989004 (94.16%) | 165594940 (86.63%) | 1222 (0.00%) | 102750070 (53.75%) | 186747556 (97.70%) | 176787559 (94.67%) | 163097252 (87.34%) | 455 (0.00%) | 100377533 (53.75%) |
| 16SHX01-00 | 72649472 (100.00%) | 67822053 (93.36%) | 61463120 (84.60%) | 278 (0.00%) | 39477814 (54.34%) | 71034840 (97.78%) | 66629371 (93.80%) | 60515551 (85.19%) | 94 (0.00%) | 38613515 (54.36%) |
| 16SHX02-00 | 77727392 (100.00%) | 73868265 (95.04%) | 68590682 (88.25%) | 221 (0.00%) | 42120434 (54.19%) | 75529802 (97.17%) | 72052676 (95.40%) | 67030870 (88.75%) | 112 (0.00%) | 40970662 (54.24%) |
| 16SHX02-01 | 96452648 (100.00%) | 91162342 (94.52%) | 84141519 (87.24%) | 717 (0.00%) | 52192873 (54.11%) | 94326999 (97.80%) | 89548232 (94.93%) | 82837748 (87.82%) | 190 (0.00%) | 51062457 (54.13%) |
| 16SHX02-02 | 213332564 (100.00%) | 201564865 (94.48%) | 185937765 (87.16%) | 1286 (0.00%) | 116009318 (54.38%) | 208545018 (97.76%) | 197919676 (94.91%) | 182984529 (87.74%) | 395 (0.00%) | 113460312 (54.41%) |
| 16ZHJ01-00 | 223665052 (100.00%) | 210943298 (94.31%) | 194300632 (86.87%) | 1079 (0.00%) | 121204796 (54.19%) | 218818403 (97.83%) | 207351330 (94.76%) | 191446532 (87.49%) | 430 (0.00%) | 118618127 (54.21%) |
| 16ZHJ01-01 | 242372132 (100.00%) | 228225811 (94.16%) | 209925129 (86.61%) | 1407 (0.00%) | 131722802 (54.35%) | 236742912 (97.68%) | 224142499 (94.68%) | 206735102 (87.32%) | 551 (0.00%) | 128694915 (54.36%) |
| 16ZHJ02-00 | 250556444 (100.00%) | 236354730 (94.33%) | 217764518 (86.91%) | 877 (0.00%) | 135407009 (54.04%) | 245054488 (97.80%) | 232265752 (94.78%) | 214509114 (87.54%) | 469 (0.00%) | 132476877 (54.06%) |
| 16ZHJ02-01 | 186643096 (100.00%) | 175783210 (94.18%) | 161734900 (86.65%) | 1063 (0.00%) | 101485453 (54.37%) | 182134216 (97.58%) | 172460491 (94.69%) | 159110171 (87.36%) | 474 (0.00%) | 99054924 (54.39%) |
| 16ZHJ03-00 | 131937596 (100.00%) | 124560896 (94.41%) | 114831160 (87.03%) | 690 (0.00%) | 71958993 (54.54%) | 129236402 (97.95%) | 122556211 (94.83%) | 113238374 (87.62%) | 404 (0.00%) | 70507880 (54.56%) |
| 16ZHJ03-01 | 165073580 (100.00%) | 155658382 (94.30%) | 143319272 (86.82%) | 614 (0.00%) | 89617049 (54.29%) | 161538679 (97.86%) | 153055396 (94.75%) | 141263893 (87.45%) | 277 (0.00%) | 87724136 (54.31%) |
| 16ZHJ03-02 | 217672084 (100.00%) | 205112329 (94.23%) | 188744663 (86.71%) | 1100 (0.00%) | 118408676 (54.40%) | 212816516 (97.77%) | 201544094 (94.70%) | 185932566 (87.37%) | 478 (0.00%) | 115808429 (54.42%) |

Additional file 1: Table S4. Statistics table of reads filter information

| **Sample** | **Clean Reads Num** | **HQ Clean Reads Num(%)** | **Read Length** | **Adapter(%)** | **Low Quality(%)** | **Poly A(%)** | **N(%)** |
| --- | --- | --- | --- | --- | --- | --- | --- |
| 16ANH01-00 | 326306 | 322788 (98.92%) | 142/142 | 15314 (9.38%) | 1725 (1.06%) | 0 (0%) | 1 (0%) |
| 16ANH02-00 | 1149812 | 1134280 (98.65%) | 142/142 | 62970 (10.96%) | 5385 (0.94%) | 0 (0%) | 12 (0%) |
| 16ANH03-00 | 539692 | 533778 (98.9%) | 142/142 | 20554 (7.62%) | 2846 (1.05%) | 0 (0%) | 2 (0%) |
| 16ANH04-00 | 1555682 | 1538306 (98.88%) | 142/142 | 50482 (6.5%) | 8622 (1.11%) | 0 (0%) | 14 (0%) |
| 16ANH05-00 | 1207446 | 1196496 (99.09%) | 142/142 | 50352 (8.34%) | 5399 (0.89%) | 0 (0%) | 4 (0%) |
| 16FJ01-00 | 986508 | 975882 (98.92%) | 142/142 | 39294 (7.96%) | 5250 (1.06%) | 0 (0%) | 10 (0%) |
| 16JL01-00 | 1268600 | 1256784 (99.07%) | 142/142 | 52322 (8.24%) | 5788 (0.91%) | 0 (0%) | 12 (0%) |
| 16JL01-01 | 2002054 | 1978080 (98.8%) | 142/142 | 62906 (6.28%) | 11901 (1.19%) | 0 (0%) | 16 (0%) |
| 16SHD01-00 | 460484 | 454694 (98.74%) | 142/142 | 30120 (13.08%) | 2752 (1.2%) | 0 (0%) | 4 (0%) |
| 16SHD01-01 | 1631734 | 1612526 (98.82%) | 142/142 | 50814 (6.22%) | 9541 (1.17%) | 0 (0%) | 12 (0%) |
| 16SHD01-02 | 1346140 | 1326936 (98.57%) | 142/142 | 41030 (6.1%) | 9567 (1.42%) | 0 (0%) | 16 (0%) |
| 16SHX01-00 | 511616 | 505638 (98.83%) | 142/142 | 18152 (7.1%) | 2945 (1.15%) | 0 (0%) | 5 (0%) |
| 16SHX02-00 | 547376 | 542640 (99.13%) | 142/142 | 37028 (13.52%) | 2297 (0.84%) | 0 (0%) | 3 (0%) |
| 16SHX02-01 | 679244 | 671754 (98.9%) | 142/142 | 25384 (7.48%) | 3689 (1.09%) | 0 (0%) | 11 (0%) |
| 16SHX02-02 | 1502342 | 1485876 (98.9%) | 142/142 | 59810 (7.96%) | 8136 (1.08%) | 0 (0%) | 19 (0%) |
| 16ZHJ01-00 | 1575106 | 1555988 (98.79%) | 142/142 | 49608 (6.3%) | 9354 (1.19%) | 0 (0%) | 14 (0%) |
| 16ZHJ01-01 | 1706846 | 1682230 (98.56%) | 142/142 | 50422 (5.9%) | 12047 (1.41%) | 0 (0%) | 19 (0%) |
| 16ZHJ02-00 | 1764482 | 1743070 (98.79%) | 142/142 | 60352 (6.84%) | 10594 (1.2%) | 0 (0%) | 9 (0%) |
| 16ZHJ02-01 | 1314388 | 1295728 (98.58%) | 142/142 | 44690 (6.8%) | 9236 (1.41%) | 0 (0%) | 13 (0%) |
| 16ZHJ03-00 | 929138 | 918732 (98.88%) | 142/142 | 29442 (6.34%) | 5159 (1.11%) | 0 (0%) | 7 (0%) |
| 16ZHJ03-01 | 1162490 | 1148240 (98.77%) | 142/142 | 35248 (6.06%) | 7085 (1.22%) | 0 (0%) | 7 (0%) |
| 16ZHJ03-02 | 1532902 | 1513488 (98.73%) | 142/142 | 49256 (6.42%) | 9630 (1.26%) | 0 (0%) | 13 (0%) |

Additional file 1: Table S5. HQ clean Reads vs. Reference Genomes

| **Sample** | **All Reads** | **Single Mapped Reads** | **Paired Mapped Reads** | **Unmapped Reads** | **Mapping Ratio(%)** |
| --- | --- | --- | --- | --- | --- |
| 16ANH01-00 | 322788 | 8689 | 136359 | 41381 | 87.18 |
| 16ANH02-00 | 1134280 | 44750 | 468259 | 153012 | 86.51 |
| 16ANH03-00 | 533778 | 21637 | 220732 | 70677 | 86.76 |
| 16ANH04-00 | 1538306 | 67107 | 631011 | 209177 | 86.40 |
| 16ANH05-00 | 1196496 | 45017 | 498716 | 154047 | 87.13 |
| 16FJ01-00 | 975882 | 34324 | 403306 | 134946 | 86.17 |
| 16JL01-00 | 1256784 | 40434 | 526544 | 163262 | 87.01 |
| 16JL01-01 | 1978080 | 80346 | 800266 | 297202 | 84.98 |
| 16SHD01-00 | 454694 | 12850 | 183128 | 75588 | 83.38 |
| 16SHD01-01 | 1612526 | 62492 | 661719 | 226596 | 85.95 |
| **16SHD01-02** | **1326936** | **63983** | **79622** | **1103709** | **16.82** |
| 16SHX01-00 | 505638 | 19407 | 206577 | 73077 | 85.55 |
| 16SHX02-00 | 542640 | 15102 | 224111 | 79316 | 85.38 |
| 16SHX02-01 | 671754 | 27593 | 272367 | 99427 | 85.20 |
| 16SHX02-02 | 1485876 | 57500 | 613365 | 201646 | 86.43 |
| 16ZHJ01-00 | 1555988 | 68374 | 629686 | 228242 | 85.33 |
| 16ZHJ01-01 | 1682230 | 71814 | 690097 | 230222 | 86.31 |
| 16ZHJ02-00 | 1743070 | 86072 | 694343 | 268312 | 84.61 |
| 16ZHJ02-01 | 1295728 | 54344 | 528752 | 183880 | 85.81 |
| 16ZHJ03-00 | 918732 | 34344 | 381903 | 120582 | 86.88 |
| 16ZHJ03-01 | 1148240 | 47867 | 465088 | 170197 | 85.18 |
| 16ZHJ03-02 | 1513488 | 66452 | 622435 | 202166 | 86.64 |

Additional file 1: Table S6. Statistical of SNP in samples’ chromosome

| **Sample** | **Clean Reads Num** | **HQ Clean Reads Num(%)** | **Read Length** | **Adapter(%)** | **Low Quality(%)** | **Poly A(%)** | **N(%)** |
| --- | --- | --- | --- | --- | --- | --- | --- |
| 16ANH01-00 | 326306 | 322788 (98.92%) | 142/142 | 15314 (9.38%) | 1725 (1.06%) | 0 (0%) | 1 (0%) |
| 16ANH02-00 | 1149812 | 1134280 (98.65%) | 142/142 | 62970 (10.96%) | 5385 (0.94%) | 0 (0%) | 12 (0%) |
| 16ANH03-00 | 539692 | 533778 (98.9%) | 142/142 | 20554 (7.62%) | 2846 (1.05%) | 0 (0%) | 2 (0%) |
| 16ANH04-00 | 1555682 | 1538306 (98.88%) | 142/142 | 50482 (6.5%) | 8622 (1.11%) | 0 (0%) | 14 (0%) |
| 16ANH05-00 | 1207446 | 1196496 (99.09%) | 142/142 | 50352 (8.34%) | 5399 (0.89%) | 0 (0%) | 4 (0%) |
| 16FJ01-00 | 986508 | 975882 (98.92%) | 142/142 | 39294 (7.96%) | 5250 (1.06%) | 0 (0%) | 10 (0%) |
| 16JL01-00 | 1268600 | 1256784 (99.07%) | 142/142 | 52322 (8.24%) | 5788 (0.91%) | 0 (0%) | 12 (0%) |
| 16JL01-01 | 2002054 | 1978080 (98.8%) | 142/142 | 62906 (6.28%) | 11901 (1.19%) | 0 (0%) | 16 (0%) |
| 16SHD01-00 | 460484 | 454694 (98.74%) | 142/142 | 30120 (13.08%) | 2752 (1.2%) | 0 (0%) | 4 (0%) |
| 16SHD01-01 | 1631734 | 1612526 (98.82%) | 142/142 | 50814 (6.22%) | 9541 (1.17%) | 0 (0%) | 12 (0%) |
| 16SHD01-02 | 1346140 | 1326936 (98.57%) | 142/142 | 41030 (6.1%) | 9567 (1.42%) | 0 (0%) | 16 (0%) |
| 16SHX01-00 | 511616 | 505638 (98.83%) | 142/142 | 18152 (7.1%) | 2945 (1.15%) | 0 (0%) | 5 (0%) |
| 16SHX02-00 | 547376 | 542640 (99.13%) | 142/142 | 37028 (13.52%) | 2297 (0.84%) | 0 (0%) | 3 (0%) |
| 16SHX02-01 | 679244 | 671754 (98.9%) | 142/142 | 25384 (7.48%) | 3689 (1.09%) | 0 (0%) | 11 (0%) |
| 16SHX02-02 | 1502342 | 1485876 (98.9%) | 142/142 | 59810 (7.96%) | 8136 (1.08%) | 0 (0%) | 19 (0%) |
| 16ZHJ01-00 | 1575106 | 1555988 (98.79%) | 142/142 | 49608 (6.3%) | 9354 (1.19%) | 0 (0%) | 14 (0%) |
| 16ZHJ01-01 | 1706846 | 1682230 (98.56%) | 142/142 | 50422 (5.9%) | 12047 (1.41%) | 0 (0%) | 19 (0%) |
| 16ZHJ02-00 | 1764482 | 1743070 (98.79%) | 142/142 | 60352 (6.84%) | 10594 (1.2%) | 0 (0%) | 9 (0%) |
| 16ZHJ02-01 | 1314388 | 1295728 (98.58%) | 142/142 | 44690 (6.8%) | 9236 (1.41%) | 0 (0%) | 13 (0%) |
| 16ZHJ03-00 | 929138 | 918732 (98.88%) | 142/142 | 29442 (6.34%) | 5159 (1.11%) | 0 (0%) | 7 (0%) |
| 16ZHJ03-01 | 1162490 | 1148240 (98.77%) | 142/142 | 35248 (6.06%) | 7085 (1.22%) | 0 (0%) | 7 (0%) |
| 16ZHJ03-02 | 1532902 | 1513488 (98.73%) | 142/142 | 49256 (6.42%) | 9630 (1.26%) | 0 (0%) | 13 (0%) |
